# Development of a Cocreated Decision Aid for Patients With Depression—Combining Data-Driven Prediction With Patients’ and Clinicians’ Needs and Perspectives: Mixed Methods Study

**DOI:** 10.2196/67170

**Published:** 2025-07-21

**Authors:** Kaying Kan, Frederike Jörg, Klaas J Wardenaar, Frank J Blaauw, Maarten F Brilman, Ellen Visser, Dennis Raven, Dwayne Meijnckens, Erik Buskens, Danielle C Cath, Bennard Doornbos, Robert A Schoevers, Talitha L Feenstra

**Affiliations:** 1 University Center for Psychiatry, Rob Giel Research Center, Interdisciplinary Center for Psychopathology and Emotion Regulation, University Medical Center Groningen, University of Groningen, PO Box 30001, Groningen, 9700 RB, The Netherlands, 31 5036161008, 31 655257786; 2 Department of Research, GGZ Friesland, Leeuwarden, The Netherlands; 3 Department of Research and Innovation, Researchable BV, Assen, The Netherlands; 4 Department of Knowledge, Innovation, and Research, MIND (Department of Knowledge, Innovation and Research), Amersfoort, The Netherlands; 5 Department of Epidemiology, University Medical Center Groningen, University of Groningen, Groningen, The Netherlands; 6 Department of Operations, Faculty of Economics and Business, University of Groningen, Groningen, The Netherlands; 7 Department of Specialist Training, GGZ Drenthe Mental Health Institute, Assen, The Netherlands; 8 Department of Science and Engineering, University of Groningen, Groningen, The Netherlands

**Keywords:** shared decision-making, clinical decision support, decision support, mental health, mental illness, mental disorder, depression, depressed, major depressive disorder, depressive disorder, precision medicine, precision care, personalized medicine, personalized care, individualized medicine, individualized care, data-driven

## Abstract

**Background:**

Major depressive disorders significantly impact the lives of individuals, with varied treatment responses necessitating personalized approaches. Shared decision-making (SDM) enhances patient-centered care by involving patients in treatment choices. To date, instruments facilitating SDM in depression treatment are limited, particularly those that incorporate personalized information alongside general patient data and in cocreation with patients.

**Objective:**

This study outlines the development of an instrument designed to provide patients with depression and their clinicians with (1) systematic information in a digital report regarding symptoms, medical history, situational factors, and potentially successful treatment strategies and (2) objective treatment information to guide decision-making.

**Methods:**

The study was co-led by researchers and patient representatives, ensuring that all decisions regarding the development of the instrument were made collaboratively. Data collection, analyses, and tool development occurred between 2017 and 2021 using a mixed methods approach. Qualitative research provided insight into the needs and preferences of end users. A scoping review summarized the available literature on identified predictors of treatment response. K-means cluster analysis was applied to suggest potentially successful treatment options based on the outcomes of similar patients in the past. These data were integrated into a digital report. Patient advocacy groups developed treatment option grids to provide objective information on evidence-based treatment options.

**Results:**

The Instrument for shared decision-making in depression (I-SHARED) was developed, incorporating individual characteristics and preferences. Qualitative analysis and the scoping review identified 4 categories of predictors of treatment response. The cluster analysis revealed 5 distinct clusters based on symptoms, functioning, and age. The cocreated I-SHARED report combined all findings and was integrated into an existing electronic health record system, ready for piloting, along with the treatment option grids.

**Conclusions:**

The collaboratively developed I-SHARED tool, which facilitates informed and patient-centered treatment decisions, marks a significant advancement in personalized treatment and SDM for patients with major depressive disorders.

## Introduction

Major depressive disorder (MDD) is a prevalent disorder that significantly impacts various aspects of life, including in the community and at home, school, and work, affecting millions of individuals globally. Despite the availability of several evidence-based treatments, such as antidepressant medication and psychotherapy [1,2], treatment responses vary significantly among patients [3]. This variability underscores the need for personalized treatment approaches to improve individual patient outcomes. One promising strategy to enhance treatment response is to predict which treatment options a patient is most likely to respond to [4], thereby reducing the trial-and-error process often associated with finding the right therapy [5]. Patients’ preferences play a crucial role in treatment outcomes, with research indicating that positive expectations regarding treatment prior to its start can enhance recovery [6].

Recently, patient empowerment has accelerated the implementation of shared decision-making (SDM). SDM is an approach where patients and clinicians make decisions together, using the best available evidence regarding screening, treatment, or management options [7]. SDM enables patient-centered choices [8,9] and is effective in achieving treatment agreement [10]. However, determining the most appropriate treatment for each patient remains challenging. SDM requires accessible information for patients and clinicians about evidence-based treatment options, including their benefits and harms [7,11-13]. In clinical practice, decision aids and feedback from routine outcome monitoring (ROM) can be valuable sources of information during the SDM process to make informed choices [14,15].

Decision aids are known to increase guideline adherence, enhance access to measurement-based care strategies, and provide personalized treatment options tailored to each patient’s characteristics and circumstances [16,17]. They also offer several additional advantages, such as increasing patients’ knowledge, improving the accuracy of risk perception, and aligning care choices with patients’ values [18]. Furthermore, decision aids reduce decisional conflict, decrease passive decision-making, and positively impact patient-clinician communication [19].

In psychiatry, ROM data are gathered systematically to monitor a patient’s progress during therapy [20]. Using feedback from ROM data may increase patient engagement in treatment [21] and positively impact treatment effectiveness, efficiency, and collaborative practice [22].

Questions arise concerning what to include in a decision aid for depression. While many biological tests, clinical observations, and patient-reported outcome measures have been found to be predictive of different MDD treatment responses, no single established measure or test has sufficient prognostic accuracy to optimally guide treatment selection [23]. A promising avenue to enhance treatment response is to facilitate informed SDM before starting treatment [24,25]. This may be achieved by identifying potentially successful treatment options and tailoring them to a patient’s clinical characteristics and preferences, initiating discussions to find the preferred option.

Existing computerized decision support (CDS) tools for patients with MDD have been developed to serve various purposes, such as facilitating screening [26], targeting specific populations (eg youth depression [27] and pregnant women with MDD [28]), supporting treatment allocation [29-31], improving treatment adherence [32], facilitating the implementation of evidence-based care [33,34], and supporting decision-making regarding pharmacological treatment [8,35-37]. Despite previous efforts, a practical CDS tool that incorporates personalized treatment recommendations based on intake information and outcome monitoring data for use in the specialized mental health care setting has, to our knowledge, not yet been developed for patients with MDD.

Therefore, this study aimed to develop an instrument for SDM in MDD through cocreation with patient representatives and in collaboration with end users (both clinicians and patients) and data scientists. The proposed “Instrument for Shared Decision-Making in Depression (I-SHARED)” CDS tool aims to provide patients and clinicians with (1) thorough, systematic information regarding symptoms, medical history, contextual factors, and potentially worthwhile treatment strategies in a digital report (patient summary) and (2) objective information regarding treatment options to guide depression treatment decisions. This study is imperative to address the variability in treatment response among patients with MDD and to enhance treatment effectiveness through personalized approaches and SDM. By developing the I-SHARED tool, the study aims to improve patient outcomes, satisfaction, and engagement in treatment. This paper reports on the development of the I-SHARED tool for use in specialized mental health care.

## Methods

### Setting

In the Northern Netherlands, a unique collaboration has been established between several specialized mental health care organizations and academic researchers [38]. This collaboration includes active client participation through client representatives and facilitates treatment innovation via applied research. Within these organizations, ROM data and health care usage data are collected prior to and during treatment. The Improving Mental Health care using Personalized treatment based on analyses of Routine data for Optimal Value and Effectiveness (IMPROVE) consortium, which includes patient representatives, researchers, a health insurer, and specialized mental health care organizations [39], created a unique joint data infrastructure called the RoQua Management Information System (RQ-MIS). This system was developed in compliance with applicable laws and regulations, including the General Data Protection Regulation (GDPR) [40]. Section A in Multimedia Appendix 1 describes the structure of data linkage via a trusted third party.

### General Procedures

The study team was co-led by 2 researchers (KK and FJ) and 2 patient representatives (DM and Paul Ulrich). Regular meetings were organized, and all major decisions regarding development and research were made collaboratively between researchers and patient representatives. The development of I-SHARED followed a mixed methods approach, comprising four phases: (1) qualitative research to understand end users’ needs, preferences, and perspectives; (2) a scoping review to identify potential predictors of treatment response; (3) the development of the I-SHARED report, which includes a patient summary of collected intake and outcome monitoring data, and the prediction of potentially successful treatment options by comparing an individual with similar patients who received treatment in the past; and (4) the development of treatment-option grids for use in clinical practice to guide the SDM process. In phase 3, routinely measured variables were identified for inclusion in the I-SHARED report, and a prediction model and graphical interface for the report were developed. The goal was to create a tool that could function independently of any specific electronic medical record system.

Mental health care usage data, ROM data, and patient characteristics were accessed via the RQ-MIS data infrastructure. Data were obtained from 2 IMPROVE-partners: the University Center of Psychiatry (UCP) and GGZ Drenthe Mental Health Institute [41]. Information regarding diagnoses, treatment types (recorded for billing purposes and registered administratively by clinicians), start and end dates of treatment, and the number and duration of treatment sessions was retrieved. The resulting dataset is referred to as the I-SHARED data.

### I-SHARED Development

#### Phase 1: Stakeholder Involvement Through Qualitative Research

In total, 3 focus group interviews were conducted with 11 patients with (a history of) depression, and 7 semistructured interviews were conducted with clinicians from 5 different mental health care organizations. The aim was to identify gaps in clinical practice, relevant components of a decision aid, preferences regarding treatment outcomes, and preferences for the user interface of the decision aid. All interviews were audio-recorded, transcribed verbatim, and analyzed using thematic content analysis [42,43]. Data collection occurred between November 2016 and June 2017 until data saturation was reached.

All interview transcripts were coded using the software package ATLAS.ti version 8.0.40.0 (ATLAS.ti Scientific Software Development GmbH). Transcripts of the focus group interviews and the semistructured interviews were first coded separately, and each perspective was compared. More details regarding the qualitative research, including recruitment, participant characteristics, data collection, and analyses, are reported elsewhere [44]. This analysis resulted in a list of proposals and preferences regarding the design and relevant input for the I-SHARED report and possible treatment outcomes.

#### Phase 2: Scoping Review

A scoping review was conducted to summarize previously identified predictors of treatment response in patients with MDD. The search was performed in September 2018 using PubMed and was restricted to papers in English. Search terms included “depression” or “depressive disorder*” in combination with “prediction,” “predictors,” “determinants,” “moderators,” “mediators,” “factors,” and “treatment outcome,” “remission,” and “response.” The scoping review identified predictors of treatment response, which were then compared with the preferences in phase 1.

#### Phase 3: I-SHARED Report Development

##### Cluster Model for Personalized Treatment Options

The I-SHARED dataset was used to develop a data-driven prediction algorithm to guide depression treatment decisions. To be included in the dataset, patients had to have a primary diagnosis of MDD (N=17,788). The dataset comprised routinely collected intake and outcome data, as well as mental health care usage data. Intake data included sociodemographic characteristics and medical and mental health information (for a complete list, see Section B in Multimedia Appendix 1). Treatment response was assessed using changes in Outcome Questionnaire-45 (OQ-45) scores during treatment [45,46]. We included individuals with at least 2 OQ-45 scores, at least 90 and at most 365 days apart during treatment. In cases with more than two measurements, the last score within 365 days was used (see Section C in Multimedia Appendix 1). Prediction modeling was based on validated Dutch OQ-45 cutoff scores to assess a clinically relevant decrease in symptoms between two measurements (reliable change index: a decrease of at least 14 points in total score) [45,47].

The health care usage data distinguished 10 types of treatment: psychotherapy, (cognitive) behavioral therapy, interpersonal therapy, family therapy, pharmacotherapy, art, dance, and movement therapies, psychomotor therapy, hospitalization, day treatment program, and a category of remaining treatments. The psychotherapy group contained treatments using techniques from various methods, in contrast to an exclusive approach such as cognitive behavioral therapy. The remaining treatment group comprised treatments that were used too infrequently to be included as a specific treatment category, such as physical therapy (eg, transcranial magnetic stimulation), physiotherapy (individual or group), and specific procedures (eg, outpatient methadone, forensic psychiatric supervision, and interpreter or sign specialist). Dummy variables were created for each patient and type of treatment to indicate if it was received between 2 OQ-45 assessments (yes or no).

In total, N=2478 patient records were suitable for the cluster analysis (see Section C in Multimedia Appendix 1 for the steps of patient selection). Table 1 presents the characteristics of this group, including the percentage of patients who showed recovery between baseline and follow-up assessment. The median duration between the first and second OQ-45 assessments was 268.5 (IQR 123) days, influenced by the choice to use the last OQ-45 score in cases with more than 2 measurements and the 90‐ to 365-day period. Information on age and sex was available for all individuals, while data on other questionnaires or sociodemographic information were often incomplete.

**Table 1.** Patient characteristics of the data used for model development.

Characteristic	Value
Number of patients	2478
Significant Recovery rate ( –Δ OQ-45^a^ >=14), n (%)	1256 (50.7)
Male, n (%)	1011 (40.8)
Baseline OQ-45 total score, mean (SD)	86.7 (23.5)
Improvement (OQ-45) points, mean (SD)	–16.5 (25.5)
Time between 2 OQ-45 measurements (days), mean (SD)	253 (79)
Type of treatment received, n (%)	
Psychotherapy	182 (7.3)
(Cognitive) behavioral therapy	570 (23)
Interpersonal therapy	203 (8.2)
Systemic therapy	124 (5.0)
Pharmacotherapy	1149 (46)
Art, dance, and movement therapies	554 (22)
Psychomotor therapy	746 (30)
Hospitalization	361 (15)
Day treatment program	92 (3.7)
Remaining treatments	920 (37)

a OQ-45: Outcome Questionnaire-45.

To inform new patients about treatment options that previously benefitted patients with similar characteristics, a cluster model was estimated in the I-SHARED dataset. Clusters were based on the 3 subscales of the OQ-45 (Symptom Distress, Interpersonal Relations, and Social Role) and age. The k-means algorithm was used for the cluster model [48]. Initially, more complex models, such as extreme gradient boosting, incorporating a range of variables, were evaluated in a prediction model. However, k-means clustering was ultimately preferred due to its lower complexity and ease of interpretation for both patients and practitioners when discussing various treatment options. Z-score normalization was first applied to the data to ensure that each subscale was equally weighted in the algorithm. To determine the optimal number of clusters, we deployed 4 techniques. First, we used an elbow plot to determine the total within-cluster sum of squared error given various cluster sizes (k). Second, we used the average silhouette width to determine the distance between clusters. Third, we used principal component analysis to evaluate the overlap between clusters [49]. Finally, we estimated the stability of clusters for each k using 100 iterations. Based on these performance measures, k was chosen to ensure a good fit, large distances between clusters, minimal overlap, and high stability. Statistical analyses were performed using RStudio IDE (version 1.4.1103) running R (version 4.0.3).

##### Sensitivity Analyses

Sensitivity analyses were performed to investigate whether different patient selection criteria would result in larger sample sizes and different distributions of treatment data. In Section F in Multimedia Appendix 1 the sample was compared with (1) a sample where the first OQ-45 measurement was within 30 days of intake instead of the main analysis in which the first OQ-45 measurement available was selected and (2) a sample where the time window of the second OQ-45 measurement was at least 60 days instead of 90 days.

##### Development of the Graphical Interface of the I-SHARED Report

Based on the outcomes of phase 1 and phase 2, items were selected for inclusion in the I-SHARED report if they were either (1) routinely captured in the data or (2) required a minimal additional administrative effort to include.

Visual feedback, including ROM results and other patient characteristics, was automatically generated for patients and clinicians from a series of applications. A generic application was built to combine the outcomes of the k-means cluster model with the generated visualizations and supporting text into a single document.

For the k-means clustering model, we implemented an OpenCPU (version 2.0.8) R-based service. Based on the answers to a series of questionnaires and the pretrained cluster model, this service can return the treatments of the reference group. To generate the visualizations in the I-SHARED report, we implemented a visualization service using the Data Driven Documents library (D3, version v5.4.0), accessed via a NodeJS web service (version 10.16.0).

**Figure 1.**
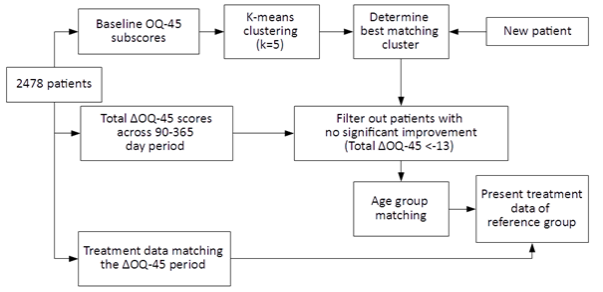
The clustering model for the presentation of treatment data of patients with a clinically relevant improvement on the OQ-45 (Outcome Questionnaire-45) total score.

The collected intake data of the individual patients were used to identify the most similar cluster. From this cluster, patients with clinically relevant improvements on OQ-45 and from similar age categories were identified to form a reference group. The age categories were <34 years old, 34-49 years old, and >50 years of age. Treatments used by patients in the reference group were extracted. Figure 1 depicts the general functioning of the algorithm. The I-SHARED report then presented the percentage of patients from the reference group who received each type of treatment. Finally, the treatment data were graphically presented in the I-SHARED report.

Following the construction of a draft version of the I-SHARED report, we conducted an additional focus group interview with 7 patients to assess the comprehensibility and added value of the visualizations in the I-SHARED report. Their feedback was used to adapt the visualizations in the final I-SHARED report, including a second treatment overview based on the cluster model. This overview now selects patients with a clinically relevant deterioration (significant increase of ≥14 points) as an alternative reference group from the relevant cluster.

### Phase 4: Treatment Option Grids

Treatment option grids were developed to meet the needs of patients with MDD in accordance with the findings from the focus group interviews. These grids were developed by MIND, a Dutch umbrella organization uniting various patient organizations involved in mental health. MIND advocates for mental health patients and their families on several important issues (eg, patient rights and quality of care), in collaboration with the Dutch Patient Association for people affected by depression (in Dutch: Depressie Vereniging). The treatment option grids reflected the evidence-based treatments advised by the national clinical guidelines for depressive disorders [50].

MIND first selected the topics and corresponding interventions relevant to patients with MDD throughout their patient journey (self-management, first-step interventions, psychotherapy, and pharmacotherapy). Second, relevant texts from the clinical guidelines were extracted on the topics. Third, new text snippets were developed to match the needs of patients with MDD. Fourth, concepts were tested by Experts by Experience from the patient association to ensure that the texts were suitable for patients. The fifth and final step included a review with the chair of the guideline development group to ensure that the new text still conformed to the clinical guidelines. After development, these option grids were field-tested along with the I-SHARED report.

### Ethical Considerations

The Medical Ethics Review Board of the University Medical Center Groningen, in accordance with the Dutch Medical Research on Human Subjects Law (in Dutch: Wet Medisch-Wetenschappelijk onderzoek met mensen,WMO), exempted the current research from full review. This waiver was granted because the study did not infringe on the physical and psychological integrity of the participants (Reference number 2017/116). Research was conducted in compliance with GDPR and Dutch privacy regulations. All participants in the qualitative study provided informed consent to participate in focus groups and individual semistructured interviews. Participants were compensated one time €25 (US $29) for the time spent in focus groups. Participants consented to the audiotaping of interviews and their use for scientific research after anonymization. Separate informed consent was obtained for the use of ROM data, or patients were given the opportunity to opt out of the use of their anonymized data in the research database. Data were anonymized and linked without personal identifiers through a trusted third party.

## Results

### Results From Qualitative Research

#### Identification of Gaps in Clinical Practice

Patients reported that a decision aid for depression could help provide a comprehensive overview of all available treatment options, including those not offered by their mental health care provider. According to patients, a decision aid that provides objective treatment advice tailored to their situation and supports SDM could help reduce clinicians’ tendency to compartmentalize.

Clinicians reported that a decision aid should ideally provide an overview of important contextual factors in addition to an overview of treatment options. It might confirm the type of treatment considered and suggest treatment options not initially thought of. They expected the decision aid to facilitate SDM, with patients being more involved and able to express their treatment preferences. Clinicians also anticipated that a data-driven decision aid could help identify profiles or clusters of patients that respond well to specific treatments, which might subsequently advance research as new data are collected and used to improve the algorithm’s performance.

#### Relevant Components of the Decision Aid

All components that patients and clinicians found relevant for inclusion in the decision aid are listed in Table 2. The final column displays components included in either the I-SHARED report or the treatment option grids. Some components were added for inclusion in future routine questionnaires (eg the Individual Recovery Outcomes Counter, Medication Adherence Rating Scale, and Mental Health Continuum-Short Form). The preferences of patients and clinicians regarding outcomes and the interface are included in the last two rows of Table 2. Along with functioning and symptom relief, the achievement of personal goals was also considered relevant by both patients and clinicians.

**Table 2.** Relevant components of the decision aid, including preferences regarding outcomes and interface.

Component		Relevant according to patients	Relevant according to clinicians	Captured in I-SHARED^a^
Depressive symptoms		✓	✓	✓
Physical complaints		✓		✓
Psychiatric comorbidities		✓	✓	✓
Personal characteristics				
Intelligence level		✓		
Coping mechanisms		✓		
Personality		✓	✓	
Physical activities		✓	✓	✓
Hobbies		✓		
Age		✓	✓	✓
Gender			✓	✓
Life events		✓		
Cause of the depression		✓	✓	
Family history of psychiatric disorders and treatment		✓	✓	✓
Contextual factors				
Patients’ own strengths and possibilities		✓		✓
Personal situation		✓		✓
Social network		✓		✓
Financial situation			✓	
Housing/relationship issues			✓	
Patient’s environment			✓	✓
Therapeutic alliance		✓		
Depression severity			✓	✓
Blood levels if applicable			✓	
Sexual complaints			✓	
Preferences regarding treatment outcomes for use in the decision aid				
Decrease of depressive symptoms		✓	✓	✓
Personal and social functioning		✓	✓	✓
Achievement of personal treatment goals		✓	✓	
Increase in quality of life			✓	✓
Chance of remission/recovery			✓	✓
Time to recurrence			✓	
Preferences regarding the interface				
Positively formulated outcomes		✓		✓
Expected outcomes of the treatment options, or overview of potentially successful treatment options		✓	✓	✓
Tailored to the individual patient		✓	✓	✓
Basic information regarding content of the treatment, goals of treatment, side effects of treatment, and treatment duration		✓		✓
A print-out or digital by email		✓	✓	✓
Discussion with the clinician/patient		✓	✓	✓
A distinction in gender and age categories when the results of the outcomes of the decision aid are displayed			✓	✓
Preferably, the expected outcomes in the data-driven analyses that take into account previous episodes, comorbidities, long-term outcomes, and the expected duration of the episode			✓	
Easy to interpret by visualizations			✓	✓

a I-SHARED: Instrument for Shared Decision-Making in Depression.

### Results of the Scoping Review

We identified 31 studies on potential predictors of treatment response in patients with depression. An overview of the studies can be found in Section D in Multimedia Appendix 1. The potential predictors were classified into four categories: (1) personal characteristics, (2) current clinical factors, (3) factors related to treatment history, and (4) biological and genetic factors. Table 3 shows the identified predictors and indicates whether they were present in current routine data and captured in I-SHARED. Predictors related to biological and genetic factors, intelligence level, income, a range of comorbidities, certain personality traits, and coping strategies were not collected routinely and therefore could not be considered for the current version of the I-SHARED report.

**Table 3.** Potential predictors of treatment response in patients with depression.

Predictors	Captured in I-SHARED^a^	Added to I-SHARED for future data collection and analysis
Personal characteristics		
Income		
Education	✓	
Marital status	✓	
Having social support		✓
Living situation	✓	
Ethnicity	✓	
(Older) age	✓	
Intelligence		
Unemployment	✓	
Current clinical factors		
Presence of psychiatric comorbidities: anxiety, bipolarity, personality disorder, and substance use disorder	✓^b^	
Current suicidal risk	✓	
Melancholic features/symptoms		
Traits: low reward dependence, low cooperativeness, high neuroticism, low extraversion, low openness, and low conscientiousness		
Depression/symptom severity	✓	
Duration of index episode	✓	
Use of medical services	✓	
Increased levels of daily hassles		
Perceived logicalness of therapy/less positive outcome expectancies/preference for treatment type		
Type of treatment	✓	
Early symptomatic improvement		
Having any significant medical comorbidity at baseline/ somatic symptoms/physical illnesses	✓	
Global functioning/executive dysfunction	✓	
Life satisfaction		✓
Self-esteem		
Psychotic features		
Increased levels of avoidance in dealing with problems		
Increased levels of dysfunctional attitudes		
Decreased levels of positive coping strategies		
Factors related to treatment history		
Nonresponse to the first antidepressant received or history of medication failure	✓	
Early onset of first depressive episode or age at onset	✓	
(High) number of previous episodes or recurrences	✓	
Lack of full remission after previous episode or more residual depressive symptomatology and psychopathology		
Higher number of hospitalizations		
Higher dosage of antidepressants	✓	
Having experienced a greater number of recent life events		
Childhood maltreatment		
Previous treatment or therapies for depression	✓	
Biological and genetic factors		
GABA^c^ levels in occipital and anterior cingulate cortices		
5-HT1A^d^ C1019 polymorphism GG genotype+A allele of BDNF^e^ G196A (Val66Met) polymorphism		
NTRK2^f^ gene polymorphisms (T-Thaplotype)		
Functional polymorphism of GRIN2B^g^		
BDNF levels at baseline		
TNF-α^h^ levels at baseline		

a I-SHARED: Instrument for Shared Decision-Making in Depression.

b Some psychiatric comorbidities are captured.

c GABA: gamma-aminobutyric acid.

d 5-HT1A: 5-hydroxytryptamine receptor subtype 1A.

e BDNF: brain-derived neurotrophic factor.

f NTRK2: neurotrophic receptor tyrosine kinase 2.

g GRIN2B: Glutamate Receptor, Ionotropic, N-Methyl-D-Aspartate, Subunit 2B.

h TNF-α: Tumor necrosis factor alpha.

### Results From the I-SHARED Report

#### Graphical Interface of the I-SHARED Report

**Figure 2.**
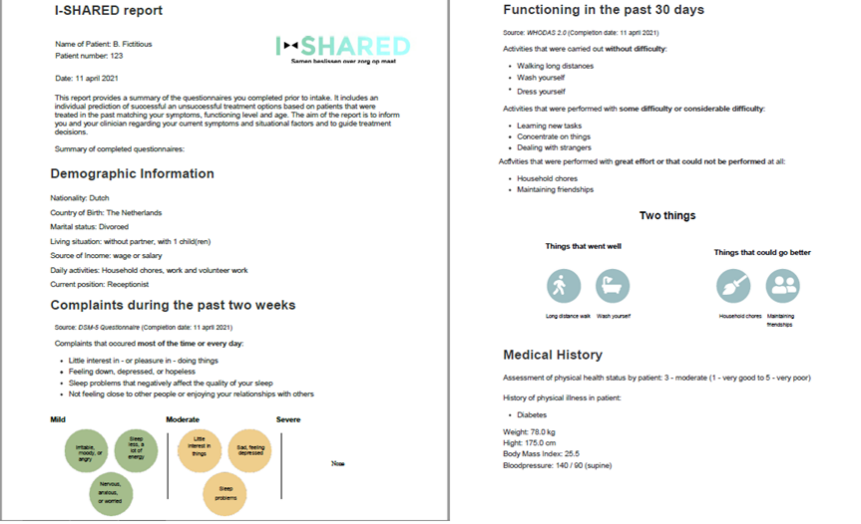
A snapshot of the graphical interface of the Instrument for shared decision-making in depression report.

A snapshot of the I-SHARED report is shown in Figure 2. Note that the original I-SHARED report was developed for national use and is therefore in Dutch. In Figure 2 data of a hypothetical patient was entered, and the report was translated into English for illustration purposes. The entire report can be printed or made available to the patient as a PDF file. Patients and clinicians discuss the content of the I-SHARED report prior to jointly deciding which treatment to initiate. The data infrastructure is designed to allow continuous improvement of the algorithm and expansion of the number of predictors in the future.

#### Cluster Modeling

**Figure 3.**
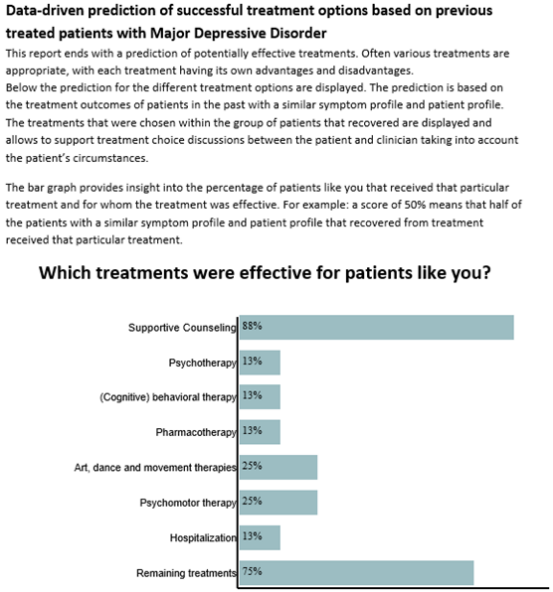
Illustration of the clustering algorithm in the I-SHARED (Instrument for Shared Decision-Making in Depression) report.

A total of 5 clusters showed the best performance, with cluster sizes ranging from 321 to 642 patients. Table 4 displays the cluster centers of the different subscales of the OQ-45. Further increasing the number of clusters did not substantially decrease the total within sum of squares errors, while the stability of clusters considerably deteriorated. Also, cluster overlap increased with the number of clusters. See Section E in Multimedia Appendix 1 for an overview of the clustered data points after applying principal component analysis. An example of the data of the clustering model as presented to the patient is shown in Figure 3. In Figure 3 data of a hypothetical patient was entered, and the information was translated into English for illustration purposes.

**Table 4.** The values of the cluster centers for the Outcome Questionnaire-45 scores subscales after reverting the z-score normalization.

Cluster	OQ-45^a^ symptom distress	OQ-45 interpersonal relations	OQ-45 social role
1	70.81	24.88	21.10
2	47.38	13.36	12.00
3	58.96	17.71	18.44
4	29.12	8.35	7.75
5	62.83	21.80	12.08
Overall mean (SD)	54.93 (15.27)	17.06 (6.55)	14.71 (5.39)

a OQ-45: Outcome Questionnaire-45 scores.

#### Sensitivity Analyses

In the sensitivity analyses, we obtained a smaller sample when selecting a first OQ-45 measurement around the time of intake (−30/+30 d). However, the distribution of treatments after intake was comparable. When the time window of the second OQ-45 measurement was at least 60 days, instead of 90 days, the sample size increased by 144 participants. Recovery rates, percentage of males, mean baseline OQ-45 score, and mean improvement during treatment were similar to the results obtained with a time window of 90‐365 days. For more details regarding the sensitivity analyses, see Section F in Multimedia Appendix 1.

#### Treatment Option Grids

Four treatment option grids were developed for patients with MDD: (1) self-management interventions, (2) short-term treatments, (3) treatment with psychotherapy and vocational therapy, and (4) treatment with pharmacotherapy. The treatment option grids provide an overview of the available evidence-based treatment options and describe when a particular treatment is used, its content, aims and side effects, and what to expect from the treatment. The treatment option grids resulted in a toolkit titled “Shared decision-making for depression - Appropriate care and support” and became publicly accessible on the Dutch national standards of mental health care website in 2021 [51].

#### The Clinical Decision Support Tool I-SHARED

The I-SHARED report, comprising the patient’s summary data and cluster-based treatment selection information, combined with the treatment option grids, resulted in the Clinical Decision Support Tool I-SHARED [41]. The tool was piloted by 2 specialized mental health care providers (results forthcoming). Clinicians were trained on how to use the personalized patient report and the treatment option grids in discussions with patients about treatment choices. This training aimed to ensure that both clinicians and patients are better informed regarding important patient and disease characteristics and potentially successful treatment options.

## Discussion

### Principal Results and Comparison With Prior Work

Using co-design and cocreation, the I-SHARED decision support tool for patients with depression was developed. I-SHARED consists of personal information summarized in a patient report, including an overview of potentially successful and unsuccessful treatment options based on reference groups, and more general information in treatment option grids. I-SHARED potentially facilitates SDM by providing patients with relevant and objective information regarding treatment options. Also, patients and health care professionals are informed about which treatments would best suit a particular patient, based on historical routine outcome data and patient (treatment) preferences.

Previous research has identified a range of patient needs to enhance SDM, including a summary of treatment options, information about potential side effects, costs and effectiveness of treatment options, examples of previous patient experiences related to the patient’s disease and treatment, discussions with their clinician, access to printed information, patient preferences and values, and information from health care professionals and health associations [11-13,52]. Several conditions need to be met to ensure that SDM becomes part of mainstream clinical practice, such as readily available evidence-based information about treatment options, guidance on weighing the pros and cons of treatment options, and a supportive clinical culture that facilitates patient engagement [7]. In our study, we began with focus group discussions to identify patient needs prior to the development of the I-SHARED tool. The needs identified by participants mostly corresponded with those identified in the studies mentioned above. Thus, most of these components were incorporated into I-SHARED or its usage, such as a supportive culture to facilitate patient-clinician discussions.

Several clinical decision support tools have been developed over the years [8,34-36]. Small study sample size hampered the predictive value of most tools regarding treatment response [23]. To address this problem, large prospective observational studies and comprehensive batteries of self-report and clinical predictors are recommended [23]. I-SHARED is based on readily available, low-cost self-report and clinical predictors data. It incorporates personalized treatment recommendations based on intake and outcome monitoring data used in the specialized mental health care setting. Several self-report questionnaires were added to I-SHARED, based on the outcomes of our qualitative research and the scoping review, to routinely capture relevant data not yet available.

In the current clustering algorithm, we used the 3 subscales of the first OQ-45 measurement. The main reason not to include other available questionnaires was lack of patients with complete data. The same was true for sociodemographic data, including living situation and education level. This is a common issue in real-world patient data. Inclusion of these variables would therefore also hinder implementation in practice. Another limiting factor was the fact that the use of less commonly measured variables would result in a model that is not easily implementable across institutions. Furthermore, results might have been influenced by the training population. To facilitate implementation across other institutions, additional training data from these institutions could be incorporated first to reduce bias within the new population. Besides, for accurate clustering, it was important to balance the number of predictors included with the number of patients available in the dataset. In future versions of the algorithm, when more patients are included in the dataset and data from additional predictors become available, we can refine predictions by adding predictors and matching filters to the clustering model.

The sensitivity analyses demonstrated that the distribution of treatments was very comparable for all options compared. Although a time window of 60‐365 days to select the second OQ-45 questionnaire resulted in a larger sample size (144 more patients), we chose the time window of 90‐365 days. This decision was made because, first, the median number of days between 2 measurements was 269 (9 mo), and second, a longer window was more likely to capture the treatment effect for psychotherapies and pharmacotherapies.

### Strengths and Limitations

A major strength of our study is the optimal use of routinely collected data prior to and during treatment in the Dutch mental health care system. The OQ-45 questionnaire was selected due to its widespread application in adult mental health care in the Netherlands and its suitability for a diverse population, thereby facilitating the potential for increased future usage of the algorithm. Although this data collection was initially set up to improve treatment monitoring, the provision of feedback on the outcomes of the questionnaires to patients is far from self-evident. By incorporating the data into the I-SHARED tool, patients and health care professionals are provided with relevant feedback for treatment selection and monitoring purposes in an accessible way. Second, the outcomes of the clustering process allowed us to inform patients and professionals about potentially successful treatment options based on historical data of treated patients with similar characteristics who had recovered after treatment. Third, the cocreation of I-SHARED by patients, patient organizations, health care professionals, and researchers resulted in a technically sound instrument appreciated by the end users. It explicitly incorporated values and preferences of both patients and professionals. By decreasing information asymmetry, both the I-SHARED report and the treatment option grids enable the patient to start a conversation with the clinician on an equal footing. In this way, I-SHARED facilitates SDM between the patient and the clinician. Patients can express their treatment preferences, and at the same time, I-SHARED provides clinicians with insight into patient-specific issues, shifting toward patient-centered care.

Our study nevertheless has several limitations. First, it was not possible to incorporate all relevant items revealed by the end users, the scoping review, and data analyses into I-SHARED. Items related to biological or genetic factors or items unknown or not recorded were omitted (eg, cause of depression and therapeutic alliance). Increasing the number of questionnaires has the disadvantage of increasing the administrative burden for patients, and some items do not lend themselves to routine monitoring and may be expensive to measure. Predictors were evaluated on overall response to treatment and not matched for the different treatment types. In addition, predictors derived from the scoping review were not weighted in importance or predictive power since we used these predictors in a cluster analysis and not in a prediction model.

Second, the use of self-report and clinical predictor data allows large sample sizes. However, after data linkage and patient selection, sample size was still moderate. This reflects mainly a lack of complete data regarding the type of treatment and outcomes during follow-up. A flexible design will allow for future updates once more complete data becomes available. Possibly, the availability of tools such as I-SHARED that allow actual use of routine data in clinical practice will enhance data completeness in the future.

Third, treatment data were derived from the treatments that were registered by clinicians for billing purposes and consequently were not always as accurate as desired. For instance, the number of unspecified follow-up contacts was relatively high. Occasionally, the registered treatment may not fully cover the precise content of the treatment received, and overlap in treatments might be possible. For example, when pharmacotherapy is registered, additional nonregistered counseling may have taken place during consultation. However, based on information about the professionals involved, a specific treatment type could be derived for most follow-up contacts. In addition, the “remaining treatments” group should ideally be disaggregated, especially for the specific group of patients that might benefit from it. The lack of specificity in this group of treatments might limit patient confidence and the decisional clarity needed for meaningful engagement.

Finally, from the patients’ feedback, we learned that those with a current depressive episode sometimes feel overwhelmed by the amount of information provided in I-SHARED. Health care professionals thus have a role in selecting the applicable treatment option grids and guiding patients through the I-SHARED report, but SDM still requires an active patient role.

### Further Research and Implications for Clinical Practice

I-SHARED focused on enhancing SDM and personalizing treatment; however, further research should investigate whether I-SHARED leads to more effective treatment allocation, improved knowledge, and decreased decisional conflict in patients with depression. Although the latter is likely to be reduced through decision aids in general, the effect on patient (mental) health and treatment effect should be further investigated [53]. In addition, we would like to expand I-SHARED by investigating the prediction of and recommendations for the type of pharmacotherapy, examining both effects and tolerability. Also, we aim to incorporate personal treatment goal formulation and monitoring into the I-SHARED report, which was not feasible in the current system.

During the pilot tests, we observed that the I-SHARED report can be used and generated for any mental disorder; however, the cluster analysis only applies to patients with depression. In its current version, the I-SHARED tool applies to patients with depression as the primary area of concern. Before the I-SHARED report can be used in other patient groups, the cluster analyses should be adapted to patients with other diagnoses, and all relevant treatment options for these diagnoses should be included.

The I-SHARED tool can deal with more recent treatment advancements and can be updated accordingly; the only requirement is that mental health care organizations must register treatment types and monitor outcomes. To date, the I-SHARED report has been implemented in several mental health care organizations and is currently being revised due to changes in questionnaire usage. When new funding becomes available, the algorithm can be updated and improved. The treatment option grids are included as a tool in the Dutch Care Standard for Depressive Disorders and are freely available on the web to inform patients regarding available and suitable treatments based on their personal preferences and goals [51]. The treatment option grids are structurally included in the cycle of revision of the Dutch Care Standard for Depressive Disorders.

I-SHARED is intended for joint use and requires training of health care professionals to use it in daily clinical practice. To this end, we developed training materials and eLearning modules. In addition, we observed that I-SHARED (and SDM in general) requires an active role from patients, who thus also need to be trained to take control during the SDM process. More information regarding I-SHARED and training materials can be found on the I-SHARED website [54].

### Conclusions

The development of the I-SHARED tool represents a significant advancement in personalized treatment and SDM for patients with MDD. By providing systematic and comprehensive information regarding symptoms, medical history, contextual factors, and treatment options, I-SHARED facilitates informed and patient-centered treatment decisions. Despite limitations, such as sample size and data completeness, the tool’s cocreation with patient representatives and collaboration with clinicians and data scientists ensures its relevance and usability in clinical practice. Future research should focus on expanding the generalizability of the tool to further enhance its usefulness in clinical practice and support impact on treatment outcomes and patient satisfaction. In addition, the effectiveness of the tool should be studied in experimental settings with a control group.

## Supplementary material

10.2196/67170 Multimedia Appendix 1 Further details of the data: variables, data infrastructure and linkage, patient selection, studies of the scoping review, and cluster and sensitivity analysis.

## Abbreviations

CDS: computerized decision support
GDPR: General Data Protection Regulation
I-SHARED: Instrument for Shared Decision-Making in Depression
IMPROVE: Improving Mental Health care using Personalized treatment based on analyses of Routine data for Optimal Value and Effectiveness
MDD: major depressive disorder
OQ-45: Outcome Questionnaire-45
ROM: routine outcome monitoring
RQ-MIS: RoQua Management Information System
SDM: shared decision-making
UCP: University Center of Psychiatry
