# Supplementary material for: Development of a Cocreated Decision Aid for Patients With Depression—Combining Data-Driven Prediction With Patients’ and Clinicians’ Needs and Perspectives: Mixed Methods Study
Source: J Particip Med. 2025 Jul 21;17:e67170. doi: 10.2196/67170 (PMC12303231; doi:10.2196/67170)
Supplement: Multimedia Appendix 1 [file jopm-v17-e67170-s001.docx]

**Section A**

**Data infrastructure and data linkage procedure**

The infrastructure, the RoQua Management Information System (RQ-MIS), enables the use of ROM and healthcare use data in scientific research projects such as IMPROVE^1^ and I-SHARED^2^ which are aimed at monitoring and improving the quality and the efficiency of care by providing clinicians and patients with meaningful and reliable information on treatment progress.

Healthcare use data and ROM data were linked using a TTP. In short, personal identifiers were pseudonymised and substantive data were encrypted at the source before being transferred to the TTP. Upon receipt by the TTP, the data were pseudonymised a second time before data from different sources were linked. Subsequently, the double pseudonymised data were encrypted by domain, i.e. converting general pseudonyms into domain-specific pseudonyms, to ensure that data from separate domains (such as different research projects) could no longer be linked. Then, the data were transferred from the TTP to the RQ-MIS where the substantive data were decrypted. The RQ-MIS operates as the custodian of the research data, and adds a final round of data-minimization and random pseudonymisation before making the data available to the researchers.

^1^ https://www.improve-nl.nl/

^2^ https://www.i-shared.nl/

**Section B**

**Overview of variables measured routinely at intake and/or during treatment**

**Personal data**

| Type of data | variables |
| --- | --- |
| Health service use and diagnosis data | Birth year  Sex  Treatment trajectory number  Start date of treatment trajectory  End date of treatment trajectory  Diagnosis according to ICD-9  Date of diagnosis  Diagnosis treatment combination (DRG) number  Start date of DRG  End date of DRG  Treatment activity (categorized in 11 treatment activities)  Treatment time spent |
| Demographics data | Marital status  Do you have children?  Do you have worries about the development or education of your children?  Does your child receive support from a care provider?  How many children do you have?  Do you have brothers or sisters?  How many brothers do you have?  How many sisters do you have?  What is your current living situation/conditions?  What is your nationality?  In which country were you born?  What is the country of birth of your biological father?  What is the country of birth of your biological mother?  What is the highest education you have completed with a diploma?  From what sources does your household receive income?  If more than 1 source: which of these is the largest source of income?  Are you currently employed?  Which of the following daily activities do you have?  Are you part of twins?  Did your biological father ever have psychiatric problems?  What psychiatric illness did your biological father have?  Has your biological father ever been treated by a psychiatrist or psychologist, or admitted to a psychiatric hospital?  Did your biological mother ever have psychiatric problems?  What psychiatric illness did your biological mother have?  Has your biological mother ever been treated by a psychiatrist or psychologist, or admitted to a psychiatric hospital?  Did your biological sibling ever have psychiatric problems?  What psychiatric illness has your biological sibling had?  Has your biological brother / sister ever been treated by a psychiatrist or psychologist, or admitted to a psychiatric hospital? |
| Substance use data | Do you smoke cigarettes, cigars, pipe or other tobacco?  At what age did you start?  How many cigarettes / cigars / pipes do you smoke on average? Per day  How often do you drink alcohol?  On a day when you drink alcohol, how many glasses do you usually drink?  Did a family member, friend, doctor, or other counsellor ever worry about your drinking or recommended that you drink less?  Have you used 1 or more of the following drugs in the past month?  How much do/did you use on a typical day? |
| Treatment history data | How old were you when you first developed psychological complaints?  How long do the current psychological complaints last?  Current and past medication use?  Have you also received psychological and / or psychotherapeutic treatments for your current complaints?  What somatic complaints do you have? |

**Validated Questionnaire data**

| World Health Organization Disability Assessment Schedule (WHODAS) |
| --- |
| DSM-5 Level 1 Cross-Cutting Symptom Measures |
| Outcome Questionnaire (OQ-45) |
| Inventory of Depressive Symptomatology self-rated (IDS-SR) |

**Section C**

**Flowchart of patient selection for clustering analysis for the prediction of treatment response**

**
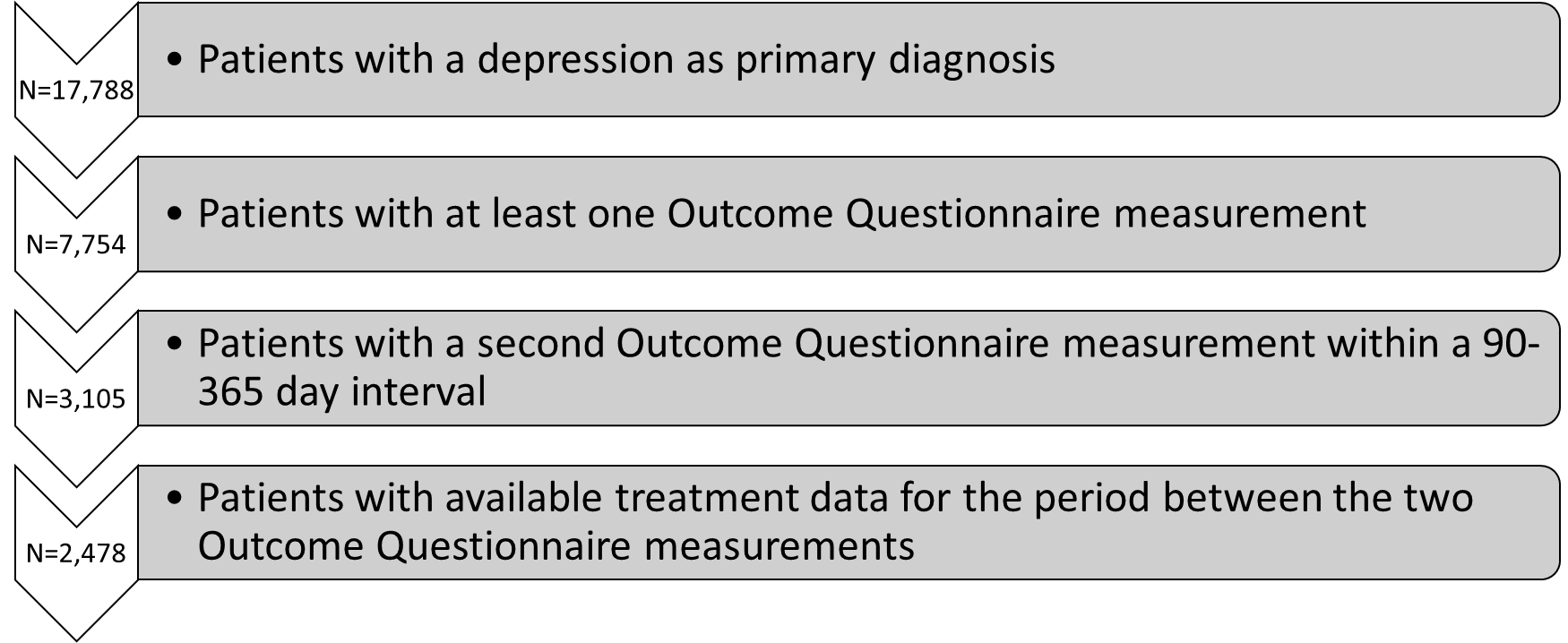
**

**Section D**

**Overview of articles derived from the literature review of potential predictors of treatment response in patients with depression**

| # | Article title and journal | Authors |
| --- | --- | --- |
| 1 | Risk factors for treatment resistance in unipolar depression: A systematic review. J Affect Disord. 2015 Jan 15;171:137-41. doi: 10.1016/j.jad.2014.09.020. Epub 2014 Oct 8. | D. Bennabi, B. Aouizerate, W. El-Hage, O. Doumy, F. Moliere, P. Courtet, I. Nieto, F. Bellivier, M. Bubrovsky, G. Vaiva, J. Holztmann, T. Bougerol, R. Richieri, C. Lancon, V. Camus, G. Saba, F. Haesbaert, T. d’Amato, T. Charpeaud, P.M. Llorca, M. Leboyer, E. Haffen |
| 2 | Severity and duration of depression, not personality factors, predict short term outcome in the treatment of major depression. J Affect Disord. 2007 Dec;104(1-3):119-26. Epub 2007 Apr 27. | Blom MB, Spinhoven P, Hoffman T, Jonker K, Hoencamp E, Haffmans PM, van Dyck R. |
| 3 | Prediction of recurrence in recurrent depression and the influence of consecutive episodes on vulnerability for depression: a 2-year prospective study. J Clin Psychiatry. 2006 May;67(5):747-55. | Bockting CL1, Spinhoven P, Koeter MW, Wouters LF, Schene AH; Depression Evaluation Longitudinal Therapy Assessment Study Group. |
| 4 | Comprehensive review of factors implicated in the heterogeneity of response in depression. Depress.Anxiety, 2012, 29, 4, 340-354, Wiley Periodicals, Inc, United States | Alatorre,C. I.; Carter,G. C.; Cantrell,R. A.; Phillips,G.; Marangell,L. B.; Goetz,I.; Victoria,Zarotsky; Haynes,V. S.; Paczkowski,R. |
| 5 | Patient predictors of response to cognitive behaviour therapy and interpersonal psychotherapy in a randomised clinical trial for depression. J.Affect.Disord., 2011, 128, 3, 252-261, Elsevier B.V, Netherlands | Carter,J.D.; Luty,S.E.; McKenzie,J.M.; Mulder,R.T.; Frampton,C.M.; Joyce,P.R. |
| 6 | Treatment Selection in Depression. Annu.Rev.Clin.Psychol., 2018, 14, 209-236, United States | Cohen,Z.D.; DeRubeis,R.J. |
| 7 | Combination psychotherapy and antidepressant medication treatment for depression: for whom, when, and how. Annu.Rev.Psychol., 2014, 65, 267-300, Annual Reviews | Craighead, W Edward; Dunlop, Boadie W |
| 8 | Personalized psychotherapy for adult depression: A meta-analytic review. Behavior Therapy, 2016, Elsevier | Cuijpers, Pim; Ebert, David D; Acarturk, Ceren; Andersson, Gerhard; Cristea, Ioana A |
| 9 | Welke psychologische behandeling, uitgevoerd door wie, is het meest effectief bij depressie? Gedragstherapie, 2010, 43, 1, 79 | Cuijpers, Pim; van Straten, Annemieke; van Oppen, Patricia; Andersson, Gerhard |
| 10 | Personalized treatment of adult depression: medication, psychotherapy, or both? A systematic review. Depress.Anxiety, 2012, 29, 10, 855-864, Wiley Periodicals, Inc, United States | Cuijpers,P.; Reynolds,C.F.,3rd; Donker,T.; Li,J.; Andersson,G.; Beekman,A. |
| 11 | Gender as predictor and moderator of outcome in cognitive behavior therapy and pharmacotherapy for adult depression: an "individual patient data" meta-analysis. Depress.Anxiety, 2014, 31, 11, 941-951, Wiley Periodicals, Inc, United States | Cuijpers,P.; Weitz,E.; Twisk,J.; Kuehner,C.; Cristea,I.; David,D.; DeRubeis,R.J.; Dimidjian,S.; Dunlop,B.W.; Faramarzi,M.; Hegerl,U.; Jarrett,R.B.; Kennedy,S.H.; Kheirkhah,F.; Mergl,R.; Miranda,J.; Mohr,D.C.; Segal,Z.V.; Siddique,J.; Simons,A.D.; Vittengl,J.R.; Hollon,S.D. |
| 12 | Socio-demographic and clinical predictors of non-response/non-remission in treatment resistant depressed patients: A systematic review. Psychiatry Res., 2016, 240, 421-430, Elsevier Ireland Ltd, Ireland | De Carlo,V.; Calati,R.; Serretti,A. |
| 13 | Predictors of remission in depression to individual and combined treatments (PReDICT): study protocol for a randomized controlled trial. Trials, 2012, 13, 106-6215-13-106, England | Dunlop,B.W.; Binder,E.B.; Cubells,J.F.; Goodman,M.M.; Kelley,M.E.; Kinkead,B.; Kutner,M.; Nemeroff,C.B.; Newport,D.J.; Owens,M.J.; Pace,T.W.; Ritchie,J.C.; Rivera,V.A.; Westen,D.; Craighead,W.E.; Mayberg,H.S. |
| 14 | Prediction of Response to Medication and Cognitive Therapy in the Treatment of Moderate to Severe Depression. J.Consult.Clin.Psychol., 2009, 77, 4, 775-787 | Fournier,J.C.; DeRubeis,R.J.; Shelton,R.C.; Hollon,S.D.; Amsterdam,J.D.; Gallop,R. |
| 15 | Cognitive behavioural therapy for depression, panic disorder and generalized anxiety disorder: a meta-regression of factors that may predict outcome. Aust.N.Z.J.Psychiatry, 2006, 40, 1, 9-19, England | Haby,M.M.; Donnelly,M.; Corry,J.; Vos,T. |
| 16 | Response of depression to electroconvulsive therapy: a meta-analysis of clinical predictors. J.Clin.Psychiatry, 2015, 76, 10, 1374-1384, Physicians Postgraduate Press, Inc, United States | Haq,A.U.; Sitzmann,A.F.; Goldman,M.L.; Maixner,D.F.; Mickey,B.J. |
| 17 | Predictive socioeconomic and clinical profiles of antidepressant response and remission. Depress.Anxiety, 2013, 30, 7, 624-630, Wiley Periodicals, Inc, United States | Jain,F.A.; Hunter,A.M.; Brooks,J.O.,3rd; Leuchter,A.F. |
| 18 | Predictors of 12-week remission in a nationwide cohort of people with depressive disorders: the CRESCEND study. Hum.Psychopharmacol., 2011, 26, 1, 41-50, England | Kim,J.M.; Kim,S.W.; Stewart,R.; Kim,S.Y.; Yoon,J.S.; Jung,S.W.; Lee,M.S.; Yim,H.W.; Jun,T.Y. |
| 19 | Prediction of treatment outcomes in psychiatry--where do we stand ? Dialogues Clin.Neurosci., 2014, 16, 4, 455-464, France | McMahon,F.J. |
| 20 | Childhood maltreatment predicts unfavorable course of illness and treatment outcome in depression: a meta-analysis. Am.J.Psychiatry, 2012, 169, 2, 141-151, United States | Nanni,V.; Uher,R.; Danese,A. |
| 21 | Moderators of outcome in late-life depression: a patient-level meta-analysis. Am.J.Psychiatry, 2013, 170, 6, 651-659, United States | Nelson,J.C.; Delucchi,K.L.; Schneider,L.S. |
| 22 | Advancing the treatment of depression with personalized medicine. J.Clin.Psychiatry, 2012, 73, 5, e17, Physicians Postgraduate Press, Inc, United States | Nierenberg,A.A. |
| 23 | Predictors of remission in the treatment of major depressive disorder: real-world evidence from a 6-month prospective observational study. Neuropsychiatr.Dis.Treat., 2015, 11, 197-205, New Zealand | Novick,D.; Hong,J.; Montgomery,W.; Duenas,H.; Gado,M.; Haro,J.M. |
| 24 | What predicts outcome, response, and drop-out in CBT of depressive adults? a naturalistic study. Behav.Cogn.Psychother., 2013, 41, 3, 365-370, United States | Schindler,A.; Hiller,W.; Witthoft,M. |
| 25 | Moderators of response in exercise treatment for depression: A systematic review. J.Affect.Disord., 2016, 195, 40-49, Elsevier B.V, Netherlands | Schuch,F.B.; Dunn,A.L.; Kanitz,A.C.; Delevatti,R.S.; Fleck,M.P. |
| 26 | Personalized medicine for depression: can we match patients with treatments? Am.J.Psychiatry, 2010, 167, 12, 1445-1455, United States | Simon,G.E.; Perlis,R.H. |
| 27 | Symptom clusters as predictors of late response to antidepressant treatment. J.Clin.Psychiatry, 2005, 66, 8, 1064-1070, United States | Trivedi,M.H.; Morris,D.W.; Grannemann,B.D.; Mahadi,S. |
| 28 | Predictors of treatment outcome in depression in later life: A systematic review and meta-analysis. J.Affect.Disord., 2018, 227, 164-182, Elsevier B.V, Netherlands | Tunvirachaisakul,C.; Gould,R.L.; Coulson,M.C.; Ward,E.V.; Reynolds,G.; Gathercole,R.L.; Grocott,H.; Supasitthumrong,T.; Tunvirachaisakul,A.; Kimona,K.; Howard,R.J. |
| 29 | Prediction of electroconvulsive therapy response and remission in major depression: meta-analysis Br.J.Psychiatry, 2018, 212, 2, 71-80, England | van Diermen,L.; van den Ameele,S.; Kamperman,A.M.; Sabbe,B.C.G.; Vermeulen,T.; Schrijvers,D.; Birkenhager,T.K. |
| 30 | Predicting the outcome of antidepressants and psychotherapy for depression: a qualitative, systematic review. Harv.Rev.Psychiatry, 2008, 16, 4, 225-234, Taylor & Francis | Van, Henricus L; Schoevers, Robert A; Dekker, Jack |
| 31 | Divergent outcomes in cognitive-behavioral therapy and pharmacotherapy for adult depression. Am.J.Psychiatry, 2016, 173, 5, 481-490, Am Psychiatric Assoc | Vittengl, Jeffrey R; Jarrett, Robin B; Weitz, Erica; Hollon, Steven D; Twisk, Jos; Cristea, Ioana; David, Daniel; DeRubeis, Robert J; Dimidjian, Sona; Dunlop, Boadie W |

**Section E: Figure with an overview of the clustered data points using 5 clusters presented in a two dimensional space using Principle Component Analysis**

**
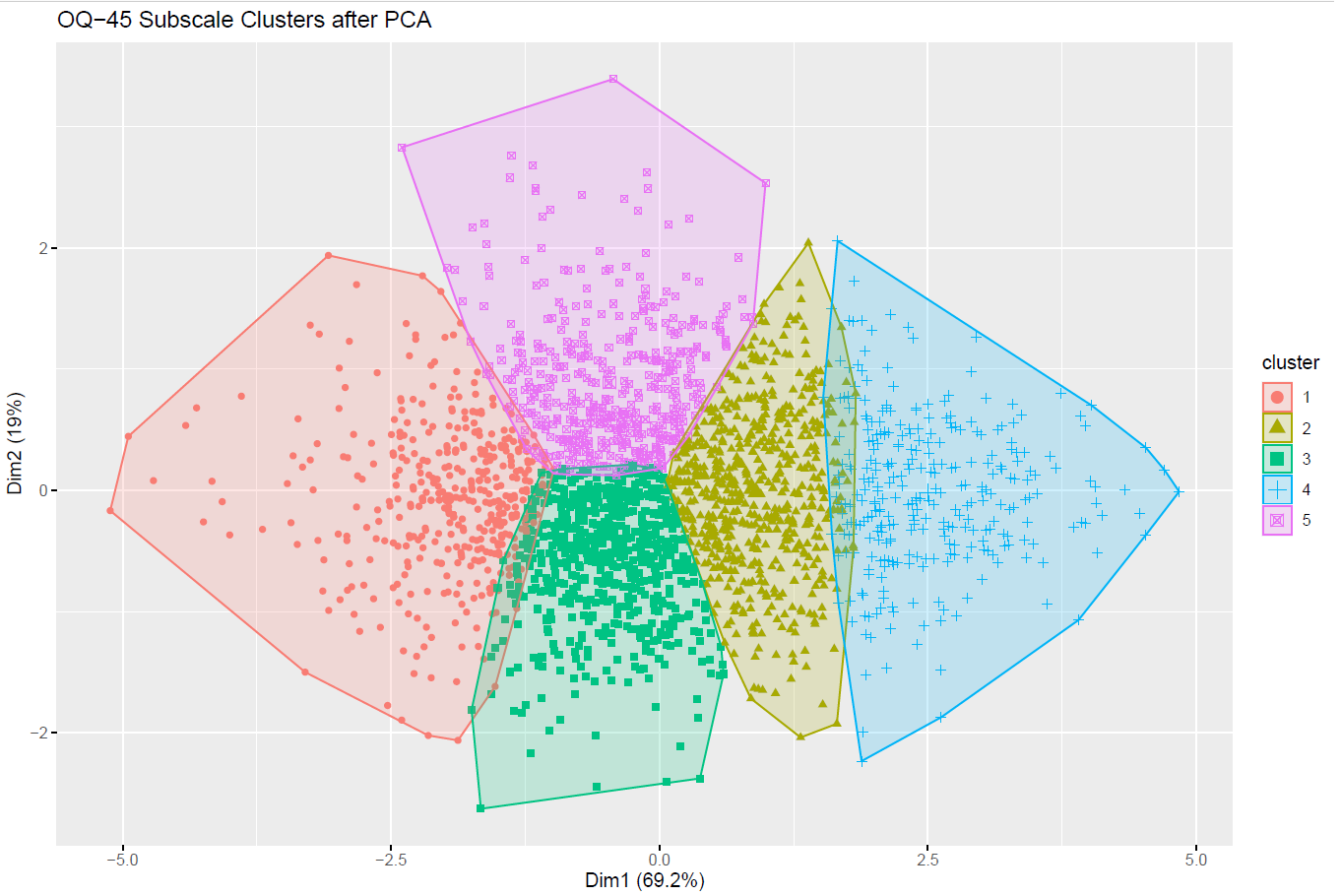
**

**Section F: Results of the Sensitivity Analysis**

**Main results in comparison with different selection criteria for the determination of the first OQ-45 measurement, adding the requirement this should be 30 days around the moment of intake**

Group 1. Main analysis: First OQ-45 taken that is available in the dataset (N=2478).
Group 2. Sensitivity analysis: First OQ-45 taken around the moment of the intake (-/+30 days) (N=1560). In both cases, patients without treatment data are removed.
In both cases, the second OQ-45 measurement is taken between 90 and 365 days after the initial one, using the last score observed within 365 days in case more than two OQ-45 scores were available.

Male (N=2478) = 40.8%, Male (N=1560) = 41.9%

| Type of treatment received | Group 1 (N=2478) | Group 2 (N=1560) |
| --- | --- | --- |
| psychotherapy  (cognitive) behavioural therapy  interpersonal therapy  systemic therapy  pharmacotherapy  art, dance and movement therapies  psychomotor therapy  hospitalization  day treatment programme  remaining treatments | 7.3%  23%  8.2%  5.0%  46%  22%  30%  15%  3.7%  37% | 6.5%  23%  10%  4.9%  45%  23%  31%  15%  3.7%  39% |

**Main results in comparison for using a time window of 60-365 rather than 90-365 days.**

| Period range (days) | 90-365 | 60-365 |
| --- | --- | --- |
| Number of patients | 2478 | 2622 |
| Significant recovery rate | 50.7% (N=1256) | 50.4% (N=1322) |
| Male | 40.8% (N=1011) | 41.0% (N=1066) |
| Mean baseline | 86.7 (SD 23.5) | 86.6 (SD 23.5) |
| Mean improvement | 16.5 (SD 25.5) | 16.5 (SD 25.4) |
